# Complete genomes of mutualistic bacterial co-symbionts “*Candidatus* Sulcia muelleri” and “*Candidatus* Nasuia deltocephalinicola” of the rice green leafhopper *Nephotettix cincticeps*

**DOI:** 10.1128/MRA.00353-23

**Published:** 2023-08-25

**Authors:** Minoru Moriyama, Yudai Nishide, Atsushi Toyoda, Takehiko Itoh, Takema Fukatsu

**Affiliations:** 1 Bioproduction Research Institute, National Institute of Advanced Industrial Science and Technology (AIST), Tsukuba, Japan; 2 Institute of Agrobiological Sciences, National Agriculture and Food Research Organization (NARO), Tsukuba, Japan; 3 Comparative Genomics Laboratory, National Institute of Genetics (NIG), Mishima, Japan; 4 School of Life Science and Technology, Tokyo Institute of Technology, Meguro-ku, Japan; 5 Department of Biological Sciences, Graduate School of Science, University of Tokyo, Bunkyo-ku, Japan; 6 Graduate School of Life and Environmental Sciences, University of Tsukuba, Tsukuba, Japan; Indiana University, Bloomington, Bloomington, Indiana, USA

**Keywords:** endosymbiont, leafhopper, bacteriome

## Abstract

The genomes of obligate bacterial co-symbionts of the green rice leafhopper *Nephotettix cincticeps*, which is notorious as an agricultural pest, were determined. The streamlined genomes of “*Candidatus* Sulcia muelleri” and “*Candidatus* Nasuia deltocephalinicola” exhibited complementary metabolic pathways for synthesizing essential nutrients that contribute to host adaptation.

## ANNOUNCEMENT

Mutualistic relationship with endocellular microbial symbionts is indispensable for growth and reproduction of diverse insect species, especially those that rely on nutritionally unbalanced diets like plant sap, vertebrate blood, and woody material (1 - 4). Many plant sap-feeding hemipteran insects belonging to the Auchenorrhyncha harbor bacteriome-associated co-primary symbionts: the ancient obligate endocellular bacterial symbiont “*Candidatus* Sulcia muelleri,” (hereafter *Sulcia*) and another microbial partner (5). Despite genome reduction, the co-symbionts retain the capability of synthesizing essential amino acids in a complementary manner, plausibly reflecting their role in nutritional supply to their plant-sucking host insects (6).

Here, we sequenced the genomes of the bacteriome-associated co-symbionts, *Sulcia* and “*Candidatus* Nasuia deltocephalinicola,” (hereafter *Nasuia*), of the rice green leafhopper *Nephotettix cincticeps* (Hemiptera: Cicadellidae) (7, 8). *N. cincticeps* is widely found in East Asia and is notorious as a serious pest of rice that vectors such pathogens as rice dwarf virus (9). *N. cincticeps* used in this study was collected from Kagoshima, Japan, and maintained on rice seedlings. The bacteriome-containing abdomens dissected from 28 adult females were subjected to DNA extraction using Genomic-tip 100/G (QIAGEN). The DNA was fragmented to an average size of 600 bp with a focused ultrasonicator M220 (Covaris). A paired-end library was constructed with a TruSeq DNA PCR-Free Library Prep Kit (Illumina) and sequenced on the Illumina HiSeq platform in a 250-bp paired-end mode. The reads derived from each symbiont were selectively retrieved by mapping to reference bacterial genomes (CP006059-CP006060) (10) using BBmap v39.01 (Bushnell B. sourceforge.net/projects/bbmap/) and samtools v1.10.2 (11). After removing low-quality reads using Trimmomatic v0.36 (12) with the following parameters: LEADING:30;TRAILING:30;MINLEN:50;ILLUMINACLIP:2:30:10, the reads recruited to the reference genomes were assembled using Unicycler v0.5.0 (13). The finalization of the genomes was manually performed with the aid of Bandage v0.8.1 (14) and IGV v2.15.4 (15). The genome annotation was carried out using DFAST pipeline v 1.2.18 (16) with manual curation.

The complete circular genomes of *Sulcia* and *Nasuia* were reconstructed from 223,209 and 23,316 paired reads, corresponding to 580-fold and 103-fold coverage, respectively. The genome of *Sulcia* was 192,344 bp with 23.7% G + C content. It encoded 186 putative protein-coding sequences (CDS), 30 transfer RNAs, and a single copy of 16S/23S/5S ribosomal RNA operon. The genome of *Nasuia* was 113,316 bp with 15.5% G + C content. It encoded 133 CDSs, 29 transfer RNAs, and a single ribosomal RNA operon. In *Nasuia*, the UGA codon was reassigned to tryptophan instead of the stop codon, as seen in other extremely reduced symbiont genomes (6).

Judging from the gene repertoire, *Sulcia* synthesizes 8 of 10 essential amino acids (Thr, Val, Leu, Ile, Lys, Phe, Trp, and Arg), while *Nasuia* is responsible for the remaining two (Met and His). It is noteworthy that methionine could be derived from the direct sulfhydrylation pathway (17). These results are consistent with prior studies highlighting the complementary nutritional roles of *Sulcia* and *Nasuia* (10, 18 - 21). Our new symbiont genome data will contribute to comparative symbiont genomic studies that provide insight into how sophisticated insect-microbe mutualisms have evolved and diversified.

## Data Availability

The sequence data have been deposited to DDBJ under the accession numbers AP028057-AP028058 for assemblies and DRR489300-DRR489301 for raw reads.
